# Performance profiles and match-to-match variability of the kinematic most demanding passages in elite men’s football: analysis of the FIFA World Cup Qatar 2022

**DOI:** 10.5114/biolsport.2026.160856

**Published:** 2026-08-24

**Authors:** Alberto Cortez, Hugo Folgado, João Brito, Farzad Yousefian, Eduardo Abade, Bruno Travassos, Bruno Gonçalves

**Affiliations:** 1 Universidade de Évora, Escola de Saúde e Desenvolvimento Humano, Departamento de Desporto e Saúde, Évora, Portugal; 2 Universidade de Évora, Comprehensive Health Research Centre (CHRC), Évora, Portugal; 3 FPF Academy, Portuguese Football Federation, Oeiras, Portugal; 4 CIPER, Faculdade de Motricidade Humana, Universidade de Lisboa; 5 Research Centre in Sports Sciences, Health Sciences and Human Development (CIDESD), Department of Sport Sciences, University of Beira Interior, Covilhã, Portugal; 6 Research Centre in Sports Sciences, Health Sciences and Human Development (CIDESD), Elite Research Community, Vila Real, Portugal; 7 Department of Sports Science, Exercise and Health, School of Life Sciences and Environment, University of Trás-os-Montes and Alto Douro, Vila Real, Portugal

**Keywords:** Analysis, Variability, Football, Profiles, Peak Periods

## Abstract

This study investigated individual performance profiles and match-to-match variability of the kinematically defined most demanding passages (MDP) during the FIFA World Cup Qatar 2022. The analysis considered different contextual factors: tournament phase (group phase vs. knockout), team ranking (top-tier vs. not-top-tier teams), match status (winning vs. not winning), and match outcome (win vs. not win), across three rolling durations (1, 3, and 5 min). A multifactorial kinematic MDP variable (MDPk) was computed as the maximal sum of the rolling average of distance covered at speeds above 15 km · h^−1^ across the defined durations and then decomposed into moderate-speed running (MSR; 15–19.8 km · h^−1^), high-speed running (HSR; 19.8–25.2 km · h^−1^), and sprinting (SPR; > 25.2 km · h^−1^). The total distance (TD) performed within the window was also considered. Cohen’s effect size analysis was used to compare players’ performance profiles and match-to-match variability. In performance profiling, trivial results were observed, with the only significant difference occurring in 1-min SPR when the team was winning (23.0 ± 19.6 m/min) compared to when it was not winning (19.6 ± 18.0 m/min) (d = 0.19 [-0.94; 1.31]). Regarding match-to-match variability, the 5-min window showed the highest variability, with all contextual factors displaying small to moderate effect sizes. This was particularly evident for match status when not winning (TD, d = 0.60 [0.90; 0.19]; MDPk, d = 0.24 [0.55; -0.17]; MSR, d = 0.38 [0.74; -0.04]; SPR, d = 0.44 [0.73; 0.06]). These findings indicate that individual MDPk may not depend on contextual factors, but match-to-match variability should be expected when MDPk is analysed across different time windows. The existence of significant variability emphasizes the necessity for individualized training approaches to optimize preparation. This underscores the importance of monitoring these passages to enhance player conditioning and improve overall performance.

## INTRODUCTION

In football, a comprehensive understanding of the physical demands placed on players is crucial for optimizing performance and enhancing the overall quality of the sport [1]. Numerous studies have explored the intricate aspects of the game, revealing the multifaceted demands imposed on players during a match [2–4]. The importance of performance profiling has been emphasized by comprehensive overviews of the physical demands endured by players during training sessions and matches [5].

External loads have been typically measured using tracking technologies, enabling researchers and practitioners to objectively quantify training and match demands, and to optimize training and competitions strategies [6]. However, recent studies have devoted their attention towards examining the most demanding passages (MDP) of match play, i.e., the maximal physical activity performed by players within a given (short) period [7–9]. It is believed that the effective preparation of players for these more intense and demanding moments might be critical not only for performance optimization but also for injury prevention [10]. Generally, the studies have identified and analysed the MDP using different time windows (e.g., 1, 3, and 5 min) using a moving-average approach [11–13]. Besides football, this method has been applied to various team sports, including rugby [14] and Australian football [15], aiming to compare match demands according to players’ position [13, 16, 17], evaluate differences between training and competition demands [18, 19], and to link high-intensity activities to players’ physical characteristics [15].

A recent study expanded upon these methodologies by incorporating a multifactorial approach to the analysis of the MDP, by using the rolling average 5-min MDP at the individual player level and considering both mechanical and kinematic aspects of match play [20, 21]. For the kinematic approach, the authors used the rolling average for distance performed above 15 km·h^−1^ and analysed these moments into their constituent variables, moderate-speed running (MSR; 15–19.8 km·h^−1^), high-speed running (HSR; 19.8–25.2 km·h^−1^), and sprinting (SPR; > 25.2 km·h^−1^), as defended by several authors [13, 22, 23]. Although different threshold definitions for HSR and sprinting have been reported in the literature [24, 25], a previous kinematic approach to MDP defined the HSR as a speed of 19.8 to 25.2 km·h^−1^ and sprinting of speed higher than 25.2 km·h^−1^ [20, 21].

However, the influence of match-specific contextual factors and the match-to-match variability of MDP_k_ and its constituent variables (MSR, HSR, and SPR) remains to be elucidated. A better understanding of each player activities over the course of a larger period can also contribute to a broader understanding of performance variability in football. This variability refers to fluctuations in physical, tactical or technical demands that players encounter during different match phases and across matches [26]. Several contextual factors have already been considered to analyse the variations in performance, including match location, quality of opposition and match outcome [27–30]. In fact, contextual factors encompass a wide range of elements, and while understanding their influence on the MDP of match play in regular competitions is essential, analysing its influence in the context of high-stakes tournaments, such as the FIFA World Cup, are crucial for elite teams (e.g., adjusting tactics and planning players’ rotations). Previous research has shown that during key tournament stages (i.e., semi-finals and finals), players present higher physical outputs [31]. Also, winning against higher-ranked opponents is associated with a greater total distance covered [32]; players cover less distance in the second half [33]; teams that draw or lose matches exhibit higher high-speed running, while lower-ranked opponents tend to accumulate higher total distance covered [34]. Although some studies have analysed data from high-stakes tournaments such as the FIFA World Cup under different contextual factors [31], research addressing match-to-match variability has predominantly focused on single teams or extended competitive periods [22, 32]. As a result, MDP remains insufficiently explored, with few studies analysing them [35, 36], while MDPk is still unexplored.

Therefore, the primary aim of the current study was to investigate the performance profile and match-to-match variability of the MDP_k_ according to various contextual factors during the FIFA World Cup Qatar 2022. The profile and individual variability of the MDP were computed using a multifactorial criterion variable (MDP_k_) and its constituent variables (MSR, HSR, and SPR). We hypothesized that the MDP_k_ is highly variable due to the influence of multiple contextual factors. Additionally, this study sought to more effectively define and analyse the kinematic MDP, thus providing novel insights into its application in performance monitoring and training optimization.

## MATERIALS AND METHODS

### Data source

Raw data from each match were downloaded from the FIFA Data Platform. The data were collected for all players across 64 matches during the FIFA World Cup Qatar 2022 using a multicamera computerized tracking system (TRACAB, Chyron Hego), with high-definition cameras operating at 25 Hz. The validity and reliability of the TRACAB systems have been previously established [37]. Goalkeepers were excluded from the analysis due to the unique positional demands of their role. For the profile, only outfield players who started the match and played at least 70 min were included; and for variability, only outfield players who started in at least two matches, each with a minimum of 70 min played, were included, resulting in a final sample of 399 players [38]. This study was conducted according to the ethical principles of the World Medical Association Declaration of Helsinki and approved by Departamento de Desporto e Saúde, Escola de Saúde e Desenvolvimento Humano, Universidade de Évora.

### Data treatment

Raw positional data were collected across all matches and processed using bespoke programming software. For all matches, data were analysed for the full match duration, including stoppage time, but excluding any extra time [39]. The player’s speed was calculated from the raw positional data, and outliers of unphysical human speeds (> 11 m/s) were replaced with the mean of adjacent data.

A multifactorial kinematic variable (MDP_k_) was computed. This variable was calculated as the maximal sum of distance covered at more than 15 km·h^−1^ considering the rolling average across the predefined durations: 1, 3 and 5 min. Subsequently, this indicator was decomposed into moderate-speed running (MSR; 15–19.8 km·h^−1^), high-speed running (HSR; 19.8–25.2 km · h^−1^), and sprinting (SPR; > 25.2 km·h^−1^), following the same procedure, but with different intensity thresholds. Finally, the maximal total distance covered (TD) within each time window was also calculated [20, 21].

The influence of four contextual factors on the MDP variables was investigated: tournament phase – group stage vs. knockout phases; team ranking, with the top 11 ranked national teams, based on the FIFA ranking (as of October 6, 2022) categorized as Top Tier and the other teams categorized as Non-Top Tier; match status, organized by winning vs. not winning (drawing or losing); and match outcome (win vs not win (draw or loss)).

The MDP_k_ was computed by applying a rolling average analysis across each match for every player over 1-, 3-, and 5-min durations, and considering the different contextual factors, generating a total of 3069 observations for 399 players.

### Statistical analysis

The match-to-match variability of MDP_k_ and its constituent variables (MSR, HSR, and SPR) and total distance (TD) were calculated using the coefficient of variation (CV) [30, 40] for each match and according to each contextual factor. Match-to-match variability was computed as within-player CV, considering only players who participated in at least two matches (ranging from 2 to 7 match observations) for each contextual factor. The variability in match performance was analysed according to the previously presented contextual factors.

Inferential analyses were carried out for both MDP_k_ result and MDP_k_ match-to-match variability, based on each contextual factor. Independent *t*-tests were used to compare within contextual factors. Additionally, estimation statistics were used to analyse the magnitude of the differences. Effect size (± 95% CI) was assessed using Cohen’ *d* and interpreted according to the following thresholds: trivial (0–0.19), small (0.20–0.49), moderate (0.50–0.79) and large (> 0.80). All statistical analyses were conducted using the Python programming language (Python 3.8) through the Spyder scientific development environment (https://www.spyder-ide.org/) and the Dabest package version 0.3.1 [41]. Statistical significance was set at a p ≤ 0.05, and all data are presented as mean ± SD, unless otherwise stated.

## RESULTS

Table 1 presents the inferential and descriptive statistics for all performance variables for the profile according to contextual factors. Following the same structure, Table 2 shows the results for match-to-match variability. Additionally, Figure 1 presents the Cohen’s d (95% confidence interval) for both profiles and variability under the considered contexts.

**TABLE 1:** Profile of MDP performance variables in each time window, considering the effect of contextual factors.

Tournament Phase Profile								

MDP Time	Variable	Group Phase	N	Knockout	N	t-statistic	p-value	Cohen’ *d* [95% CI]
1	TD	189.3 ± 25.4 m/min	766	189.8 ± 23.8 m/min	257	-0.32	0.75	0.02 [-0.12; 0.16]
1	MDPk	109.5 ± 22.3 m/min	766	109.6 ± 22.0 m/min	257	-0.02	0.99	0.00 [-0.14; 0.14]
1	MSR	51.0 ± 21.2 m/min	766	51.1 ± 20.2 m/min	257	-0.01	0.99	0.00 [-0.14; 0.14]
1	HSR	38.2 ± 16.5 m/min	766	38.0 ± 16.3 m/min	257	0.16	0.88	-0.01 [-0.15; 0.13]
1	SPR	20.3 ± 18.8 m/min	766	20.5 ± 17.4 m/min	257	-0.16	0.88	0.01 [-0.13; 0.15]
3	TD	147.4 ± 17.6 m/min	766	147.6 ± 17.4 m/min	257	-0.18	0.86	0.01 [-0.13; 0.15]
3	MDPk	60.1 ± 12.5 m/min	766	59.5 ± 12.6 m/min	257	0.68	0.50	-0.05 [-0.19; 0.09]
3	MSR	32.3 ± 10.9 m/min	766	31.5 ± 10.2 m/min	257	0.99	0.32	-0.07 [-0.21; 0.07]
3	HSR	19.4 ± 7.5 m/min	766	19.5 ± 7.5 m/min	257	-0.17	0.87	0.01 [-0.13; 0.15]
3	SPR	8.4 ± 7.6 m/min	766	8.4 ± 6.8 m/min	257	-0.06	0.95	0.00 [-0.14; 0.15]
5	TD	137.3 ± 15.8 m/min	766	135.6 ± 15.1 m/min	257	1.57	0.12	-0.11 [-0.25; 0.03]
5	MDPk	48.7 ± 10.7 m/min	766	47.5 ± 10.4 m/min	257	1.62	0.11	-0.12 [-0.26; 0.03]
5	MSR	27.1 ± 8.3 m/min	766	26.4 ± 8.0 m/min	257	1.24	0.22	-0.09 [-0.23; 0.05]
5	HSR	15.5 ± 5.5 m/min	766	15.1 ± 5.5 m/min	257	1.00	0.32	-0.07 [-0.21; 0.07]
5	SPR	6.1 ± 5.0 m/min	766	6.0 ± 4.8 m/min	257	0.30	0.76	-0.02 [-0.16; 0.12]

**Ranking Profile**								

**MDP Time**	**Variable**	**Top Tier**	**N**	**Not Top Tier**	**N**	**t-statistic**	**p-value**	**Cohen’ *d* [95% CI]**

1	TD	189.2 ± 24.0 m/min	426	189.5 ± 25.7 m/min	597	-0.21	0.84	0.01 [-0.11; 0.14]
1	MDPk	109.4 ± 22.0 m/min	426	109.6 ± 22.4 m/min	597	-0.17	0.87	0.01 [-0.11; 0.13]
1	MSR	51.9 ± 21.1 m/min	426	50.4 ± 20.8 m/min	597	1.10	0.27	-0.07 [-0.19; 0.05]
1	HSR	37.6 ± 16.4 m/min	426	38.6 ± 16.4 m/min	597	-0.96	0.34	0.06 [-0.06; 0.19]
1	SPR	19.9 ± 18.7 m/min	426	20.6 ± 18.2 m/min	597	-0.60	0.55	0.04 [-0.09; 0.16]
3	TD	147.0 ± 17.8 m/min	426	147.8 ± 17.4 m/min	597	-0.78	0.44	0.05 [-0.07; 0.17]
3	MDPk	59.7 ± 12.7 m/min	426	60.1 ± 12.5 m/min	597	-0.45	0.65	0.03 [-0.10; 0.15]
3	MSR	32.1 ± 10.9 m/min	426	32.1 ± 10.7 m/min	597	0.04	0.97	0.00 [-0.13; 0.12]
3	HSR	19.2 ± 7.3 m/min	426	19.6 ± 7.6 m/min	597	-0.81	0.42	0.05 [-0.07; 0.18]
3	SPR	8.4 ± 7.5 m/min	426	8.4 ± 7.4 m/min	597	-0.01	0.99	0.00 [-0.12; 0.12]
5	TD	136.2 ± 15.8 m/min	426	137.3 ± 15.6 m/min	597	-1.07	0.28	0.07 [-0.06; 0.19]
5	MDPk	48.1 ± 10.6 m/min	426	48.5 ± 10.6 m/min	597	-0.61	0.54	0.04 [-0.09; 0.16]
5	MSR	26.6 ± 8.2 m/min	426	27.1 ± 8.2 m/min	597	-1.07	0.28	0.07 [-0.06; 0.19]
5	HSR	15.6 ± 5.4 m/min	426	15.3 ± 5.6 m/min	597	0.63	0.53	-0.04 [-0.16; 0.08]
5	SPR	6.0 ± 4.7 m/min	426	6.1 ± 5.1 m/min	597	-0.23	0.82	0.01 [-0.11; 0.14]

**Match Status Profile**								

**MDP Time**	**Variable**	**Winning**	**N**	**Not Winning**	**N**	**t-statistic**	**p-value**	**Cohen’ *d* [95% CI]**

1	TD	192.0 ± 24.5 m/min	218	188.7 ± 25.1 m/min	805	1.76	0.08	-0.13 [-0.28; 0.02]
1	MDPk	111.9 ± 22.8 m/min	218	108.9 ± 22.0 m/min	805	1.75	0.08	-0.14 [-0.29; 0.01]
1	MSR	51.2 ± 21.3 m/min	218	51.0 ± 20.9 m/min	805	0.12	0.91	-0.01 [-0.16; 0.14]
1	HSR	37.7 ± 16.0 m/min	218	38.3 ± 16.5 m/min	805	-0.46	0.64	0.03 [-0.11; 0.18]
1	SPR	23.0 ± 19.6 m/min	218	19.6 ± 18.0 m/min	805	2.31	0.02 *	-0.19 [-0.34; -0.04]
3	TD	148.8 ± 17.9 m/min	214	147.1 ± 17.4 m/min	809	1.24	0.22	-0.10 [-0.25; 0.05]
3	MDPk	60.2 ± 12.3 m/min	214	59.9 ± 12.6 m/min	809	0.26	0.80	-0.02 [-0.17; 0.13]
3	MSR	32.3 ± 10.7 m/min	214	32.0 ± 10.8 m/min	809	0.37	0.71	-0.03 [-0.18; 0.12]
3	HSR	19.3 ± 7.6 m/min	214	19.5 ± 7.4 m/min	809	-0.31	0.76	0.02 [-0.13; 0.17]
3	SPR	8.5 ± 7.5 m/min	214	8.4 ± 7.4 m/min	809	0.21	0.83	-0.02 [-0.17; 0.13]
5	TD	136.7 ± 15.3 m/min	196	136.9 ± 15.8 m/min	827	-0.18	0.86	0.01 [-0.14; 0.17]
5	MDPk	48.6 ± 10.2 m/min	196	48.3 ± 10.7 m/min	827	0.42	0.68	-0.03 [-0.19; 0.12]
5	MSR	26.8 ± 7.7 m/min	196	26.9 ± 8.3 m/min	827	-0.28	0.78	0.02 [-0.13; 0.18]
5	HSR	15.6 ± 5.8 m/min	196	15.4 ± 5.4 m/min	827	0.55	0.58	-0.05 [-0.20; 0.11]
5	SPR	6.3 ± 4.9 m/min	196	6.0 ± 5.0 m/min	827	0.68	0.50	-0.05 [-0.21; 0.10]

**Match Outcome Profile**								

**MDP Time**	**Variable**	**Win**	**N**	**Not Win**	**N**	**t-statistic**	**p-value**	**Cohen’ *d* [95% CI]**

1	TD	190.0 ± 25.1 m/min	393	189.0 ± 25.0 m/min	630	0.57	0.57	-0.04 [-0.16; 0.09]
1	MDPk	110.3 ± 22.8 m/min	393	109.1 ± 21.8 m/min	630	0.86	0.39	-0.06 [-0.18; 0.07]
1	MSR	51.9 ± 21.5 m/min	393	50.5 ± 20.7 m/min	630	0.96	0.34	-0.06 [-0.19; 0.06]
1	HSR	37.4 ± 16.3 m/min	393	38.6 ± 16.5 m/min	630	-1.10	0.27	0.07 [-0.06; 0.20]
1	SPR	21.0 ± 19.2 m/min	393	19.9 ± 17.9 m/min	630	0.90	0.37	-0.06 [-0.18; 0.07]
3	TD	147.2 ± 17.2 m/min	393	147.6 ± 17.8 m/min	630	-0.37	0.71	0.02 [-0.10; 0.15]
3	MDPk	60.0 ± 12.3 m/min	393	60.0 ± 12.7 m/min	630	0.01	0.99	0.00 [-0.13; 0.13]
3	MSR	31.8 ± 10.6 m/min	393	32.3 ± 10.8 m/min	630	-0.72	0.47	0.05 [-0.08; 0.17]
3	HSR	19.5 ± 7.4 m/min	393	19.4 ± 7.5 m/min	630	0.16	0.87	-0.01 [-0.14; 0.12]
3	SPR	8.7 ± 7.8 m/min	393	8.2 ± 7.2 m/min	630	0.88	0.38	-0.06 [-0.18; 0.07]
5	TD	136.7 ± 15.4 m/min	393	137.0 ± 15.9 m/min	630	-0.31	0.75	0.02 [-0.11; 0.15]
5	MDPk	48.4 ± 10.5 m/min	393	48.4 ± 10.7 m/min	630	0.05	0.96	0.00 [-0.13; 0.12]
5	MSR	26.9 ± 8.3 m/min	393	26.9 ± 8.2 m/min	630	-0.03	0.98	0.00 [-0.12; 0.13]
5	HSR	15.3 ± 5.5 m/min	393	15.5 ± 5.5 m/min	630	-0.44	0.66	0.03 [-0.10; 0.15]
5	SPR	6.2 ± 5.1 m/min	393	6.0 ± 4.9 m/min	630	0.62	0.53	-0.04 [-0.17; 0.09]

* p ≤ 0.05; Abbreviations: TD: total distance; MDPk: kinematic MDP performance variable; MSR, moderate-speed running; HSR: high-speed running; SPR: sprinting; N: sample size per condition; CI: confidence intervals.

**TABLE 2:** Variability of MDP performance variables in each time window, considering the effect of contextual factors.

Tournament Phase Variability								

MDP Time	Variable	Group Phase	N	Knockout	N	t-statistic	p-value	Cohen’ *d* [95% CI]
1	TD	8.9 ± 7.0 %	246	8.7 ± 5.7 %	63	0.32	0.75	-0.04 [-0.32; 0.24]
1	MDPk	12.0 ± 7.6 %	246	13.3 ± 9.2 %	63	-1.01	0.31	0.16 [-0.12; 0.44]
1	MSR	32.4 ± 21.5 %	246	35.3 ± 20.6 %	63	-0.98	0.33	0.14 [-0.14; 0.41]
1	HSR	36.0 ± 22.6 %	246	35.2 ± 21.5 %	63	0.26	0.79	-0.04 [-0.31; 0.24]
1	SPR	87.0 ± 44.6 %	246	75.6 ± 45.1 %	63	1.78	0.08	-0.26 [-0.54; 0.02]
3	TD	7.2 ± 5.0 %	246	6.3 ± 3.9 %	63	1.52	0.13	-0.19 [-0.46; 0.09]
3	MDPk	10.8 ± 7.2 %	246	12.4 ± 9.0 %	63	-1.27	0.21	0.20 [-0.07; 0.48]
3	MSR	23.2 ± 14.8 %	246	27.0 ± 15.5 %	63	-1.78	0.08	0.26 [-0.02; 0.53]
3	HSR	29.3 ± 18.6 %	246	28.3 ± 15.7 %	63	0.43	0.66	-0.06 [-0.33; 0.22]
3	SPR	79.6 ± 43.8 %	246	72.0 ± 36.5 %	63	1.41	0.16	-0.18 [-0.46; 0.10]
5	TD	6.4 ± 4.1 %	246	5.4 ± 3.6 %	63	2.02	0.05 *	-0.26 [-0.54; 0.02]
5	MDPk	11.0 ± 6.8 %	246	11.2 ± 7.7 %	63	-0.23	0.82	0.04 [-0.24; 0.31]
5	MSR	18.8 ± 11.9 %	246	22.0 ± 12.3 %	63	-1.86	0.07	0.27 [-0.01; 0.55]
5	HSR	24.2 ± 14.7 %	246	25.5 ± 16.6 %	63	-0.56	0.57	0.09 [-0.19; 0.36]
5	SPR	67.6 ± 41.1 %	246	73.6 ± 40.8 %	63	-1.04	0.30	0.15 [-0.13; 0.42]

**Ranking Variability**								

**MDP Time**	**Variable**	**Top Tier**	**N**	**Not Top Tier**	**N**	**t-statistic**	**p-value**	**Cohen’ *d* [95% CI]**

1	TD	7.6 ± 5.0 %	100	7.5 ± 5.3 %	180	0.19	0.85	-0.02 [-0.27; 0.22]
1	MDPk	11.5 ± 6.2 %	100	9.8 ± 5.7 %	180	2.30	0.02 *	-0.29 [-0.54; -0.05]
1	MSR	27.6 ± 15.3 %	100	26.1 ± 15.3 %	180	0.78	0.44	-0.10 [-0.34; 0.15]
1	HSR	31.6 ± 16.4 %	100	27.6 ± 17.1 %	180	1.95	0.05 *	-0.24 [-0.48; 0.00]
1	SPR	74.2 ± 34.7 %	100	65.2 ± 34.5 %	180	2.07	0.04 *	-0.26 [-0.51; -0.01]
3	TD	6.0 ± 4.2 %	100	5.8 ± 3.4 %	180	0.31	0.76	-0.04 [-0.29; 0.20]
3	MDPk	10.6 ± 5.5 %	100	8.4 ± 5.3 %	180	3.35	0.00 *	-0.42 [-0.67; -0.18]
3	MSR	21.2 ± 10.5 %	100	18.3 ± 11.1 %	180	2.16	0.03 *	-0.26 [-0.51; -0.02]
3	HSR	25.1 ± 13.3 %	100	23.5 ± 13.8 %	180	0.99	0.32	-0.12 [-0.37; 0.12]
3	SPR	62.5 ± 35.7 %	100	62.9 ± 32.3 %	180	-0.10	0.92	0.01 [-0.23; 0.26]
5	TD	5.2 ± 3.2 %	100	5.2 ± 3.1 %	180	0.01	0.99	0.00 [-0.25; 0.24]
5	MDPk	10.3 ± 5.3 %	100	8.6 ± 5.3 %	180	2.61	0.01 *	-0.33 [-0.57; -0.08]
5	MSR	16.7 ± 8.2 %	100	14.7 ± 9.1 %	180	1.89	0.06	-0.23 [-0.47; 0.02]
5	HSR	21.9 ± 11.3 %	100	20.1 ± 11.2 %	180	1.25	0.21	-0.16 [-0.40; 0.09]
5	SPR	55.7 ± 30.0 %	100	55.7 ± 33.6 %	180	0.00	1.00	0.00 [-0.24; 0.24]

**Match Status Variability**								

**MDP Time**	**Variable**	**Winning**	**N**	**Not Winning**	**N**	**t-statistic**	**p-value**	**Cohen’ *d* [95% CI]**

1	TD	8.9 ± 7.9 %	44	9.4 ± 6.7 %	242	-0.40	0.69	0.07 [-0.25; 0.40]
1	MDPk	11.5 ± 8.7 %	44	12.8 ± 8.1 %	242	-0.99	0.33	0.17 [-0.15; 0.49]
1	MSR	33.9 ± 21.6 %	44	33.2 ± 20.4 %	242	0.19	0.85	-0.03 [-0.35; 0.29]
1	HSR	32.4 ± 22.1 %	44	34.4 ± 23.1 %	242	-0.53	0.60	0.08 [-0.24; 0.41]
1	SPR	81.5 ± 43.6 %	44	85.1 ± 46.5 %	242	-0.49	0.63	0.08 [-0.25; 0.40]
3	TD	6.1 ± 4.9 %	36	7.3 ± 4.1 %	238	-1.42	0.16	0.29 [-0.06; 0.64]
3	MDPk	10.0 ± 7.7 %	36	10.9 ± 6.9 %	238	-0.71	0.48	0.14 [-0.21; 0.49]
3	MSR	24.6 ± 18.4 %	36	23.4 ± 14.2 %	238	0.39	0.70	-0.08 [-0.43; 0.27]
3	HSR	26.7 ± 20.2 %	36	28.9 ± 18.3 %	238	-0.61	0.54	0.12 [-0.23; 0.47]
3	SPR	64.3 ± 46.2 %	36	77.2 ± 43.6 %	238	-1.56	0.13	0.30 [-0.06; 0.65]
5	TD	4.1 ± 3.3 %	24	6.6 ± 4.2 %	245	-3.41	0.00 *	0.60 [0.18; 1.02]
5	MDPk	9.8 ± 6.0 %	24	11.6 ± 7.2 %	245	-1.31	0.20	0.24 [-0.18; 0.66]
5	MSR	14.6 ± 10.7 %	24	19.1 ± 11.9 %	245	-1.91	0.07	0.38 [-0.04; 0.80]
5	HSR	22.0 ± 14.8 %	24	24.5 ± 14.9 %	245	-0.78	0.44	0.17 [-0.25; 0.58]
5	SPR	50.5 ± 31.9 %	24	68.8 ± 42.5 %	245	-2.60	0.01 *	0.44 [0.02; 0.86]

**Match Outcome Variability**								

**MDP Time**	**Variable**	**Win**	**N**	**Not Win**	**N**	**t-statistic**	**p-value**	**Cohen’ *d* [95% CI]**

1	TD	9.2 ± 7.3 %	91	9.1 ± 7.2 %	192	0.10	0.92	-0.01 [-0.26; 0.24]
1	MDPk	13.1 ± 8.0 %	91	11.9 ± 8.1 %	192	1.08	0.28	-0.14 [-0.39; 0.11]
1	MSR	33.8 ± 21.0 %	91	32.3 ± 20.0 %	192	0.55	0.58	-0.07 [-0.32; 0.18]
1	HSR	36.1 ± 23.9 %	91	34.7 ± 23.3 %	192	0.48	0.63	-0.06 [-0.31; 0.19]
1	SPR	83.5 ± 47.8 %	91	84.1 ± 46.6 %	192	-0.10	0.92	0.01 [-0.24; 0.26]
3	TD	7.4 ± 5.0 %	91	7.2 ± 5.3 %	192	0.24	0.81	-0.03 [-0.28; 0.22]
3	MDPk	11.4 ± 6.7 %	91	10.4 ± 6.8 %	192	1.21	0.23	-0.15 [-0.40; 0.10]
3	MSR	23.8 ± 13.9 %	91	22.4 ± 15.7 %	192	0.71	0.48	-0.09 [-0.34; 0.16]
3	HSR	27.8 ± 18.8 %	91	29.7 ± 19.7 %	192	-0.78	0.44	0.10 [-0.15; 0.35]
3	SPR	69.5 ± 39.0 %	91	78.0 ± 46.3 %	192	-1.59	0.11	0.19 [-0.06; 0.44]
5	TD	5.8 ± 4.7 %	91	6.6 ± 4.4 %	192	-1.50	0.14	0.20 [-0.05; 0.45]
5	MDPk	11.5 ± 7.3 %	91	10.0 ± 6.9 %	192	1.62	0.11	-0.21 [-0.46; 0.04]
5	MSR	17.7 ± 11.3 %	91	17.8 ± 13.0 %	192	-0.08	0.93	0.01 [-0.24; 0.26]
5	HSR	25.9 ± 16.0 %	91	23.8 ± 15.2 %	192	1.03	0.30	-0.13 [-0.38; 0.12]
5	SPR	67.2 ± 38.4 %	91	67.9 ± 43.7 %	192	-0.13	0.90	0.02 [-0.23; 0.26]

* p ≤ 0.05; Abbreviations: TD: total distance; MDPk: kinematic MDP performance variable; MSR, moderate-speed running; HSR: high-speed running; SPR: sprinting; N: sample size per condition; CI: confidence intervals.

**FIG. 1:**
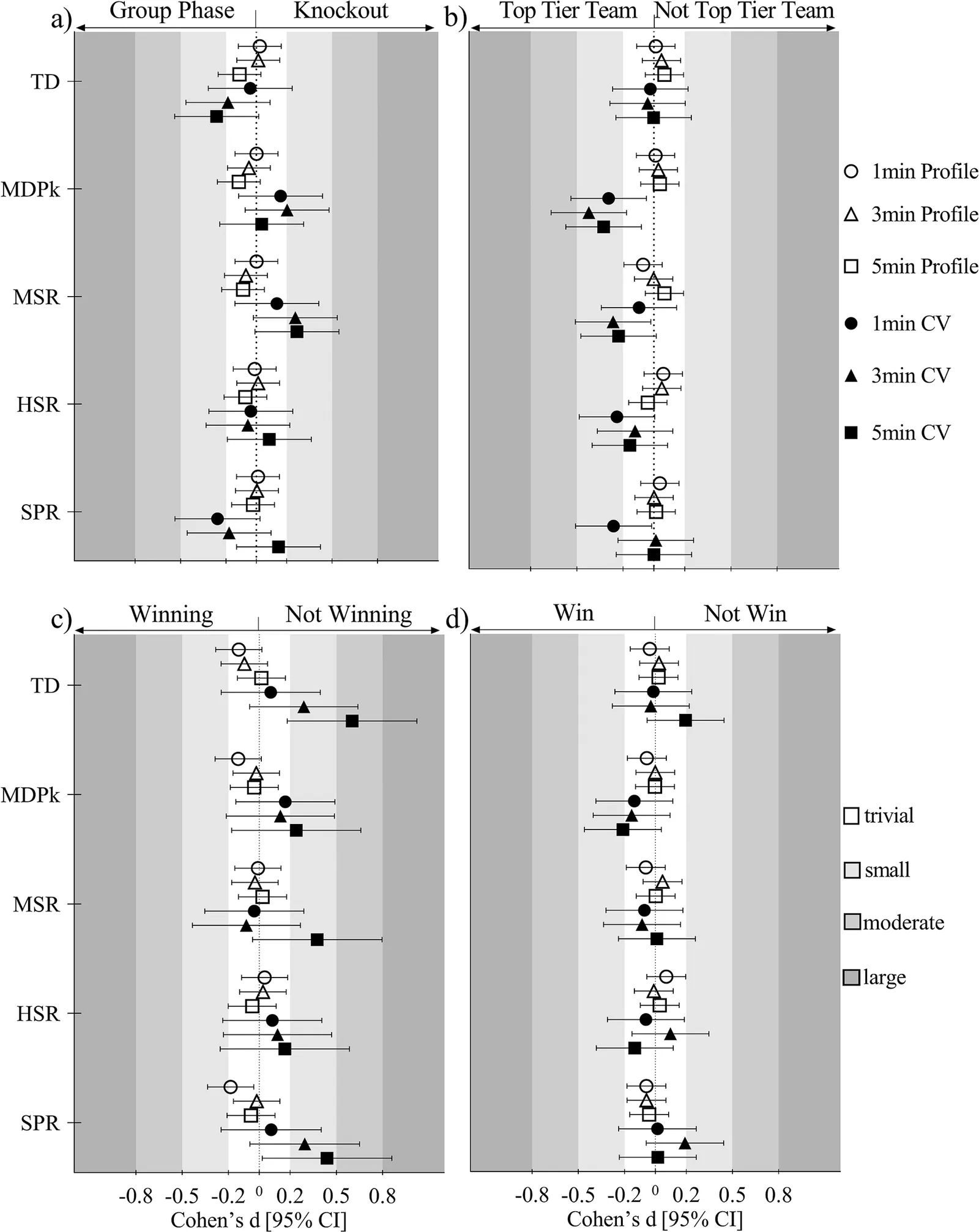
Effect size of the variables using Cohen’s d (95% confidence interval) for both variability and profiles under different contexts: a) tournament phase; b) ranking; c) match status; d) match outcome.

The results showed a decrease over larger time windows (from 1, to 3 and 5 min) across all performance variables, highlighting the impact of interval duration on player movement intensity. Accordingly, considering the 1-min interval, players covered 189.4 ± 25.0 m/min for TD, 109.5 ± 22.2 m/min for the MDP_k_ distance, and for each component of MDP_k_, 51.0 ± 20.0 m/min for MSR, 38.2 ± 16.4 m/min for HSR, and 20.3 ± 18.4 m/min for SPR. A reduction in distances was observed in the 3-min time window, with players covering 147.5 ± 17.5 m/min in TD, 60.0 ± 12.6 m/min in MDP_k_, and for each component of MDP_k_, 32.1 ± 10.7 m/min in MSR, 19.5 ± 7.5 m/min in HSR, 8.4 ± 7.4 m/min in SPR. This continued at the 5-min interval, where the average distances were further reduced to 136.9 ± 15.7 m/min for TD, 48.4 ± 10.6 m/min for MDP_k_, and for each component of MDP_k_, 26.9 ± 8.2 m/min in MSR, 15.4 ± 5.5 m/min in HSR, and 6.1 ± 5.0 m/min in SPR.

In general, players’ profiles did not exhibit any significant differences across the specific contextual factors, except for 1-min SPR. For this variable, players covered greater distances while winning compared to not winning (t = 2.31; p = 0.02; *d* = 0.19 [-0.94;1.31]).

Regarding variability, in the tournament phase, the results indicate trivial to small effects. Higher variability in the group phase for 1-min SPR (t = 1.78; p = 0.08; d = -0.26 [0.03; -0.54]) and 5-min TD (t = 2.02; p = 0.05; d = -0.26 [0.03; -0.48]) was observed (Table 2). In contrast, during the knockout phase, the MSR exhibited higher variability in both 3-min (t = -1.78; p = 0.08; d = 0.26 [0.55; -0.01]) and 5-min windows (t = -1.86; p = 0.07; d = 0.27 [0.56; -0.01]).

Regarding team ranking, the top-tier teams exhibited slightly higher variability across all time windows in MDP_k_, including 1-min (t = 2.30; p = 0.02; d = -0.29 [-0.05; -0.55]), 3-min (t = 3.35; p = 0.00; d = -0.42 [-0.18; -0.68]), and 5-min (t = 2.61; p = 0.01; d = -0.33 [-0.08; -0.59]) windows (Table 2). A similar trend was also observed for the other performance variables, with top-tier teams consistently showing greater variability in the high-intensity variables and in the shorter time windows, particularly 1-min HSR (t = 1.95; p = 0.05; d = -0.24 [0.00; -0.49]) and 1-min SPR (t = 2.07; p = 0.04; d = -0.26 [-0.02; -0.51]). Additionally, MSR had higher variability in the 3-min (t = 2.16; p = 0.03; d = -0.26 [-0.03; -0.53]) and 5-min (t = 1.89; p = 0.06; d = -0.23 [0.00; -0.48]) windows.

According to the match status, teams that were not winning exhibited a small to moderate tendency for higher variability. This was particularly evident in the 3-min TD (t = -1.42; p = 0.16; d = 0.29 [0.67; -0.15]) and SPR (t = -1.56; p = 0.13; d = 0.30 [0.65;-0.09]) (Table 2). In the 5-min window, HSR showed a trivial effect (t = -0.78; p = 0.44; d = 0.17 [0.56; -0.25]) but small effects were observed for MDP_k_ (t = -1.31; p = 0.20; d = 0.24 [0.55;-0.17]), MSR (t = -1.91; p = 0.07; d = 0.38 [0.74; -0.04]), and SPR (t = -2.60; p = 0.01; d = 0.44 [0.73; 0.06]). Notably, 5-min TD (t = -3.41; p = 0.00; d = 0.60 [0.90; 0.19]) exhibited moderately higher variability when not winning compared to teams that were winning.

For match outcome, the results displayed trivial effects across all the variables, with two exceptions observed in the 5-min TD (t = -1.50; p = 0.14; d = 0.20 [0.44;-0.09]) and MDP_k_ (t = 1.62; p = 0.11; d = -0.21 [0.04;-0.47]), both of which demonstrated a small effect (Table 2).

## DISCUSSION

The present study investigated the effect of different contextual factors on players’ performance profile and match-to-match variability of kinematic MDP variables across three time window durations. To our knowledge, this study is the first to analyse the profiles and variability of the MDP according to multifactorial MDP performance variables. The results showed that the contextual factors investigated did not significantly influence the profile of any of the performance variables studied. However, different match-to-match variability was observed according to team ranking and match status, with 1-min high-intensity metrics (HSR and SPR) in top-tier teams and 5-min TD, MDP_k_, MSR, and SPR when not winning revealing greater effect size variability.

The analysis of MDP in three distinct intervals identified an inverse relationship between interval duration and MDP intensity. Such results are consistent with previous research demonstrating a decline in relative intensities as the duration of the analysed time window increased [12, 42].

Other key findings indicate that the profile of the MDP remains consistent regardless of contextual factors, which suggests that the physical and physiological demands placed on players during these critical periods are inherent to the nature of the game itself, rather than being influenced by external factors. This consistency in MDP profiles underscores the importance of preparing players to handle these intense passages uniformly, irrespective of the specific match context. Interestingly, our results showed similar values for TD in the 1-min window (~189 m · min^−1^) compared to those reported in other studies for TD in the peak period (~188 m · min^−1^) [43]. Thus, the 1-min MDP_k_ values align closely with previously reported peak period metrics, supporting the reliability and validity of our findings.

To assess match-to-match variability across contextual factors, the coefficient of variation (CV) was calculated for players’ TD, MDP_k_, MSR, HSR and SPR. Overall, the results indicate higher variability of players’ MDP considering shorter time windows and during high-intensity displacements. The match-to-match variability of the relative TD was found to be stable across all moving average durations (CV: 4.0–9.4%). This aligns with previous research quantifying match-to-match variability of TD covered (CV: 6.8–7.3%) in elite football, which has been reported to be relatively low [38]. Similarly, prior research has indicated that TD covered remains largely unchanged under different contextual factors, including environmental conditions, level of opposition, and match outcome [44, 45]. As a result, the main differences in physical performance are likely better reflected in fluctuations in HSR and SPR, as these variables exhibit considerably higher match-to-match variability (HSR CV: ~16–38%; SPR CV: ~21–76%) [10]. In the present study, match-to-match variability of the relative HSR (CV: 20.1–36.1%) and SPR (CV: 50.5–87.0%) showed a similar trend.

For the first time, MDP_k_ match-to-match variability was also analysed in relation to contextual factors. Interestingly, top-tier teams exhibited greater variability in HSR and SPR within 1-min windows. This variability can be attributed to the frequent adaptation of strategies and tactics based on the opponent and match context, leading to fluctuations in high-intensity activities throughout the tournament [46]. Additionally, the depth and quality of the squad in top-tier teams facilitate player rotation, which allow coaches to strategically manage player workloads as the tournament progresses [47]. Also, the match status (not winning) exhibited high variability in 5-min windows for TD, MDP_k_, MSR and SPR. These performance fluctuations, when teams that are not winning, may lead to a higher frequency of intense efforts to respond to the evolving match situation, regain possession, and create scoring opportunities [48]. Similarly, the high variability observed during not winning scenarios suggests an adaptive behaviour, as players must continuously adjust their positioning and intensity to change the score [18]. Regarding match outcome, the data suggest that player variability is not related to it, supporting the premise that external load metrics often function independently of the final scoreline [49]. The greater variability associated with high-intensity movements highlights the collective nature of performance fluctuations. Additionally, because HSR and SPR represent a smaller proportion of TD, even minor variations between Profile and variability of the most demanding passages matches result in greater relative variability. Given this sensitivity, strategies, positions, roles, and tactical approaches could also contribute to variability in high-intensity actions.

Notably, this study comprehensively analysed match-to-match variability in kinematic MDP within team sports. Unlike previous research, this study examined match-to-match variability across three rolling average windows (1, 3 and 5 min), rather than a single duration (3 min) [10]. Thus, it is the first to report changes in match-to-match variability for the kinematic MDP performance metrics using a multifactorial criterion variable and its respective constituent variables [20, 21]. Furthermore, this study focused exclusively on starting players who completed at least 70 min per match, minimizing the potential influence of substitution on the MDP [50]. However, we acknowledge that the dataset was collected from a single tournament, and certain influencing factors were not analysed. Furthermore, previous research has shown that playing positions exhibit significantly different physical demands [43, 51, 52]. Additional contextual factors, such as environmental conditions, time of season, and temporal distribution, have also been found to influence physical outputs [49, 53, 54]. Therefore, future research should consider these factors when analysing the match-to-match variation in kinematic MDP performance to provide a more holistic understanding of performance variability in elite football.

Practitioners regularly use total match data to prepare players or competition, and in this regard, MDP should not be overlooked. While it has been argued that relying solely on MDP data to inform training may only prepare athletes for the average MDP demands, analysing the variability of the metric can provide a more specific target range for the training drills, allowing for a more individualized and adaptive approach to performance preparation.

## CONCLUSIONS

Our findings suggest that, overall, the relative intensities and variability in MDP performance, across three durations, exhibit trivial to small effects in relation to the selected contextual factors when playing positions were not considered. This suggests that these contextual factors are likely modulated by position-specific demands, which should be considered in future research at an individual level in order to understand the individuality of each player according to the requirements of each position and team. Additionally, this study provides valuable insights into the variability of MDP during match play by quantifying variance in MDP analyses, which is essential for contextualizing performance data and thereby enhancing ecological validity and practical applicability. Such contextualization allows coaches to differentiate MDP fluctuations at the individual level, enabling more precise comparisons of physical performance across matches and supporting data-driven decision-making in training and competition planning.
